# From adolescence to old age: how sensory precision shapes body ownership during physiological aging

**DOI:** 10.3389/fnhum.2025.1663505

**Published:** 2025-10-09

**Authors:** Isabella Martinelli, Gaia Risso, Tommaso Bertoni, Valentina Meregalli, Enrico Collantoni, Franco Molteni, Alessandra Pedrocchi, Gabriella Bottini, Andrea Serino, Michela Bassolino

**Affiliations:** ^1^Department of Brain and Behavioral Sciences, University of Pavia, Pavia, Italy; ^2^School of Health Sciences, HES-SO Valais-Wallis, Sion, Switzerland; ^3^The Sense Innovation and Research Center, Sion/Lausanne, Switzerland; ^4^Department of Neurosciences, University of Padua, Padua, Italy; ^5^MySpace Lab, Department of Clinical Neurosciences, University Hospital Lausanne (CHUV), Lausanne, Switzerland; ^6^Padua Neuroscience Center, University of Padua, Padua, Italy; ^7^Villa Beretta Rehabilitation Center, Valduce Hospital Como, Costa Masnaga, Italy; ^8^Department of Electronics, Information and Bioengineering, Politecnico di Milano, Milan, Italy; ^9^Cognitive Neuropsychology Center, ASST Grande Ospedale Metropolitano Niguarda, Milan, Italy

**Keywords:** body ownership, body representations, multisensory integration, proprioception, sensory precision, aging, older adult, Bayesian Causal Inference

## Abstract

Body ownership relies on the integration of multisensory signals coming from the environment and the body itself. Considering the substantial neurophysiological and sensory modifications occurring across the lifespan, this study aims to quantitatively evaluate age-related changes in hand ownership and its underlying bottom-up sensory and top-down components from adolescence to advanced aging. Ninety-two healthy women aged 15–83 underwent a virtual-reality based visuo-proprioceptive disparity task in which they performed reiterative reaching movements towards visual targets while observing a virtual-hand that could be spatially congruent or displaced at different disparities from the real hand’s position. Ownership was assessed by collecting reaching errors (implicit) and asking ownership judgments toward the virtual-hand (explicit). Errors were modeled using a Bayesian Causal Inference framework in which ownership for the virtual-hand resulted from a weighted average between pure visual and pure proprioceptive guidance according to their relative precision (i.e., bottom-up sensory components), and to the a priori probability that the virtual-hand was one’s own (i.e., top-down prior). Results showed that both explicit and implicit ownership towards spatially incongruent virtual-hands was higher with advancing age. Moreover, the sensory components extracted from the model revealed higher proprioceptive and lower visual variability in older adults, suggesting that as proprioception declines, visual input increasingly assumes a dominant role. No age-effect was found on the prior (i.e., top-down component). We concluded that ownership progressively changes from adolescence to old age, mostly driven by a physiological reduction in proprioceptive abilities. The sensory recalibration toward visual reliance might reflect a compensatory mechanism to maintain coherent body ownership despite age-related sensory decline.

## 1 Introduction

Body Representations (BRs) have been defined as patterns of neural coding that track the state of the body in time and space, arising from the continuous integration of multisensory signals coming from the environment and the body itself (Bassolino and Serino, 2022). BRs are extremely plastic, being shaped by interactions and adapting online to sensorimotor experiences.

Evidence has demonstrated transient alterations of BRs in healthy subjects, for instance after multisensory training (Bruno and Bertamini, 2010), the use of tools (Martel et al., 2016), or through the administration of experimental paradigms leading to some classical illusions such as the rubber hand (RHI), in which the synchronous stroking of a visible rubber hand and of the real, hidden hand, leads subjects to experience the rubber hand as part of their own body (Botvinick and Cohen, 1998). Long-term changes are evident after a brain damage, as in the case of stroke patients showing several BRs-related disorders (Serrada et al., 2025; Vallar and Ronchi, 2009; Bassolino et al., 2022). This also includes alterations in the sense of body ownership (BO) i.e., “the feeling that a given body part belongs to us” (Romano and Maravita, 2019).

On the other hand, BRs may also be impacted by the natural, physiological changes occurring during development and aging (Kállai et al., 2017; Raimo et al., 2021; Weijs et al., 2021), known to affect the physical body-structure, the functioning of the sensory and motor systems and the capacity to integrate multisensory information (Murray et al., 2016).

From a developmental perspective, several studies have shown that multisensory processing of bodily stimuli unfolds over a prolonged period and is intricately linked to the profound physical transformations the body undergoes from infancy through late childhood (de Klerk et al., 2021). As an example, studies suggest that the effect of the RHI is stronger in 4–9 years-old children compared to adults, suggesting that at this age BO is more influenced by visual cues from the rubber hand than proprioceptive information of one’s own hand (Cowie et al., 2013; Filippetti and Crucianelli, 2019). After this age, the sensitivity to the illusion normalizes at the adult level. Further evidence supporting the influence of age on BRs comes from studies by Martel et al. (2016, 2021), who demonstrated that the plastic changes in upper-limb perceived dimensions elicited by tool use, widely documented in adults (Bruno et al., 2019; Canzoneri et al., 2013; Galigani et al., 2020), manifest differently across developmental stages with the typical adult-like pattern emerging only at late puberty (Martel et al., 2021).

If development is characterized by a progressive maturation of sensory capacities, aging is marked by their decline (Adamo et al., 2007; Goble et al., 2009; Murray et al., 2016) with possible implications for BRs (Kuehn et al., 2018; Sorrentino et al., 2021). Previous studies applying the RHI on young and older adults have shown conflicting evidence. For instance, Graham et al. (2015) reported age-related effects on the illusion, with a decrease in subjective ownership ratings and an increase in proprioceptive drift among the elderly. Conversely, Marotta et al. (2018) found that both young and older adults experienced stronger subjective ownership for the rubber hand compared to middle-aged individuals, with no differences in the proprioceptive drift across age-groups. Finally, Palomo et al. (2018) suggested no age-related changes in the RHI. Overall, these conflicting results could arise from variations in the experimental protocols, intrinsic limitations in the RHI paradigm, and limited sample size.

More recently, novel experimental paradigms have advanced the study of BO and the underlying multisensory processing.

Inspired by a neurophysiological study in monkey (Fang et al., 2019), Bertoni et al. (2023) developed a virtual-reality (VR) based Visuo-Proprioceptive Disparity task (VPD) in which participants perform reaching movements toward a visual target while they see a virtual hand moving in an either spatially congruent or incongruent manner with respect to the position of the real (non-visible) hand. Importantly, the degree of visuo-proprioceptive disparity varies at each trial to avoid sensorimotor adaptations (Henriques and Cressman, 2012; Krakauer, 2009). In this paradigm, BO is measured implicitly by reaching errors and explicitly by subjective ownership ratings toward the virtual hand. Interestingly, the proprioceptive and visual contributions underlying ownership can be modeled through the application of a Bayesian Causal Inference (Bayesian CI) model. Results in healthy subjects showed significant correlation between the two measures of ownership, with both reaching errors and explicit ownership ratings varying as a function of spatial disparity, and higher reported ownership at lower disparity. Crucially, the model demonstrated that reduced proprioceptive precision led to a higher tendency to experience ownership towards the virtual hand (Bertoni et al., 2023; Mastria et al., 2025).

Recently, Risso et al. (2025) applied this paradigm to assess whether BO differed in two groups of healthy older and young adults. Findings demonstrated a higher tendency to embody the virtual hand both at the implicit (reaching error) and explicit (subjective ratings) level in the aged group. Moreover, the model revealed that the changes in ownership observed in older adults were mostly driven by a decrease of proprioceptive precision in that group (Risso et al., 2025). While these results allow us to characterize the contribution of proprioception in age-related changes in BO, these are limited to the comparison of two well-separated age-group, i.e., young (mean age: 27.17 ± 4.27) versus older (mean age: 73.82 ± 6.36) adults, lacking generalizability to other ages.

On the other hand, Weijs et al. (2024) proposed a mixed-reality task in which participants observe their own hand together with temporally mismatching multimodal stimuli, providing explicit ownership ratings and visuo-motor/tactile synchrony judgments. Interestingly, authors used a life-span approach by administering the same experimental paradigm from children to older adults (aged 7–80) and investigating the continuous effect of age on explicit ownership and the underlying multimodal processes. Results of this approach showed that with increasing age participants reported higher explicit ownership ratings across all multisensory mismatches. However, this study lacks in evaluating changes in implicit ownership and in proposing a model to explain the multisensory mechanisms underlying these observed changes.

Extending the approach from Risso et al. (2025) and Weijs et al. (2024), the current study aims at evaluating the trajectory of BO and the underlying multisensory processing during the physiological aging from adolescence to old age (15–83 years), through the administration of the VPD paradigm and the use of a Bayesian CI model in a large cohort of participants (*N* = 92). In line with Risso et al. (2025), we predict age-related changes in ownership resulting from a physiological reduction in proprioceptive precision occurring from young to old age.

## 2 Methods

### 2.1 Participants

Ninety-two healthy females aged 15–83 years were recruited. All participants were right-handed as assessed by the Flanders Handedness survey (Nicholls et al., 2013), had corrected-to-normal vision and presented no psychiatric nor neurological deficits and no pain nor sensorimotor pathologies in the upper-limbs. Recruitment and data collection were conducted across multiple experimental sites by trained experimenters. The study protocol was approved by the local ethics committees at each participating site (CER-VD, project identifier 2017-01588; Politecnico di Milano Ethical Committee, approval 11/2024; Hospital of Vicenza Ethical Committee, approval 1831). A subset of data including 15 older participants (aged 65–83) and 11 young participants (aged 22–38) has been previously published elsewhere (Risso et al., 2025).

### 2.2 Experimental procedure

While immersed in a VR-environment using a head-mounted display and a motion controller, participants underwent the VPD battery consisting of a multisensory reaching task to evaluate ownership and three complementary, (mainly) unisensory tasks including a proprioceptive judgment task, an open loop reaching task and a midline judgment task, *ad hoc* designed to independently test relevant parameters (i.e., proprioceptive and visual precision) part of the Bayesian CI model. The right, dominant hand was tested in all subjects. As data collection took place at multiple experimental sites, different VR-systems were used (i.e., Oculus Rift S, HTC-Vive, Pico), all operating the same custom-developed software implemented in Unity. A brief description of each task is provided below (for an extensive explanation, see Bertoni et al., 2023). Concerning the complementary tasks, note that according to Héroux et al. (2022, 2024), these can be considered as targeting high-level proprioceptive capacities as they involve proprioceptive judgments made in different frames of reference (i.e., processing of hand/body signals and of visual targets localization, each with their own coordinate system).

#### 2.2.1 Visuo- Proprioceptive Disparity task (VPD)

**FIGURE 1:**
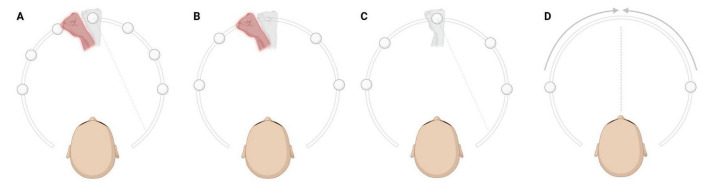
Behavioral tasks. The real, proprioceptive hand, hidden from the view of participants, is represented in light gray. The virtual hand is represented here in red (see that in virtual-reality (VR), it was displayed in natural skin color). Finally, the visual targets are represented by the white spheres. **(A)** Visuo-Proprioceptive Disparity task (VPD). Participants performed reaching movements toward one of seven equally spaced targets. At each trial, the spatial congruency between the position of participants’ real hand and the position of the virtual hand was manipulated by introducing various degrees of disparities in random order. The figure represents an example of a trial in which a negative disparity was applied, resulting in a leftward displacement of the virtual hand. **(B)** Proprioceptive Judgement task (PJ). The experimenter passively moved the participants’ real, hidden hand to one of five possible positions indicated by the white spheres (which were not visible to the participants). In the VR environment, the virtual hand appeared either to the left (as illustrated here) or to the right of the participants’ real hand, and subjects were then asked to verbally indicate whether the virtual hand was positioned to the left or to the right of their real (hidden) hand in a two-alternative forced-choice task converging algorithm. **(C)** Open Loop Reaching task (OLR). Participants had to reach visual targets relying solely on proprioception, i.e., by following the position of their real hand in the absence of any visual feedback. **(D)** Midline Judgement task (MJ). Participants were asked to report when a moving visual cue was perceived as aligned with their perceived body midline (not visible). The cue could either move from left to right, or from right to left. Created in BioRender. Martinelli, I. (2025) https://BioRender.com/pgmjc5y.

As described elsewhere (Bertoni et al., 2023; Mastria et al., 2025; Risso et al., 2025), in the VPD task participants were asked to perform reiterative reaching movements directed towards one of seven possible visual targets (white spheres) positioned along an arc and equally spaced between −45° and +45° relative to the participants’ body midline. At each trial, the spatial congruency between the position of participants’ real hand and the virtual hand observed in VR was manipulated by randomly introducing various degrees of disparities. A 0° disparity indicated visuo-proprioceptive congruency, meaning that the visual virtual hand was spatially aligned with the position of the real hand. Negative disparities (i.e., −13.3°, −20.0°, −26.6° or −40°) consisted in counterclockwise rotations of the virtual hand with respect to the real hand’s position, resulting in a leftward displacement (as in Figure 1A). Conversely, positive disparities (i.e., +13.3°, +20.0°, +26.6° or +40°) consisted in clockwise rotations, leading to a rightward displacement. The task included three experimental blocks assessing ownership under implicit and explicit conditions. Proxies of implicit ownership were collected by measuring the reaching errors, defined as the distance between the real hand position at the end of the reaching movement and the target position. A correction of systematic biases in reaching, possibly due to tracking or VR calibration, was done by subtracting at the individual level the mean reaching bias at zero spatial disparity:


$$N\,o\,r\,m\,a\,l\,i\,z\,e\,d\,E\,r\,r\,o\,r_{i}=O\,b\,s\,e\,r\,v\,e\,d\,E\,r\,r\,o\,r_{i}-\frac{1}{N_{s\,u\,b\,j\,e\,c\,t,\alpha =0}}\,\sum_{j\epsilon \left\{subject,\alpha =0\right\}}O\,b\,s\,e\,r\,v\,e\,d\,E\,r\,r\,o\,r_{j}$$

Explicit ownership was assessed during the last block by asking subjective ratings of ownership toward the virtual hand, using a scale from 1 to 10.

#### 2.2.2 Bayesian Causal Inference model and its application to the VPD task

Recent studies provided evidence that BO emerges from a Bayesian Inference process based on the optimal integration of multisensory inputs (Bertoni et al., 2023; Chancel et al., 2022; Fang et al., 2019). During the VPD task, reaching toward a target is achieved by integrating visual information of the incongruent virtual hand with proprioceptive information of the real hand’s position. As proposed by previous works on healthy subjects (Bertoni et al., 2023; Risso et al., 2025) and stroke patients (Mastria et al., 2025), the reaching performance at this task can be effectively modeled within a Bayesian CI framework which posits that the endpoint reaching position results from a weighted average of visual and proprioceptive inputs according to their precision (variabilities σ_*P*_ for proprioception, σ_*V*_ for vision), and to the a priori probability that the visual and the proprioceptive information have a common cause (P_*com prior*_). Importantly, higher variability corresponds to lower precision and decreases the reliability of a sensory modality. Further information on the Bayesian CI model, including its probabilistic formulation, is outlined in the Supplementary material.

#### 2.2.3 Proprioceptive Judgment task (PJ)

The PJ was a proprioceptive task under passive condition. During PJ, the experimenter passively moved the participants’ real hand to one out of five possible target positions along an arc, equally spaced between −45° and +45° relative to the participants’ body midline. At each trial, a virtual hand spatially displaced with respect to the real hand’s position appeared, and participants had to report whether it was perceived at the right or left of their real hidden hand (Figure 1B). A two forced-choice adaptive algorithm was used to iteratively select the next stimulus based on participants’ responses, progressively converging toward the perceived hand position. Proprioceptive precision (σ_PJ_), corresponding to the variability in the perceived hand localization and proprioceptive accuracy (PJ absolute error), corresponding to the absolute angular difference between the perceived and the real hand position along the trials, were computed from this task.

#### 2.2.4 Open Loop Reaching task (OLR)

The OLR task was a proprioceptive task implying active reaching movements. The procedure for the OLR was similar to that of the VPD, as participants had to perform reaching movements towards one out of five visual targets (white sphere) positioned along an arc, equally spaced between −45° and +45° from the body midline. While immersed in VR, participants received no visual feedback about their hand localization (i.e., no virtual hand, nor any other hand-related visual cue) and thus relied entirely on continuous proprioceptive information about their limb throughout the task. However, they were provided with visual information about the target to reach, as in the VPD task (Figure 1C). Two measures were computed from this task: proprioceptive precision (σ_OLR_), corresponding to the variability in the proprioceptive reaching bias, and proprioceptive accuracy (OLR absolute error), corresponding to the angular difference between the target position and the real hand position along the different trials, in absolute value.

#### 2.2.5 Midline Judgement task (MJ)

The MJ task was a visuo-proprioceptive alignment task in which a white sphere moved horizontally across the participants’ field of view at a speed of 10°/s, starting from ± 45°, ± 40°, ± 35°, and ± 30° relative to the participants’ body midline (negative starting angles indicate that the white sphere moved from the left visual field to the right, while positive starting angles that the sphere moved from the right to the left). At each trial, participants had to verbally report when they felt that the visual cue was aligned with the perceived and not visible midline of their body (Figure 1D). This task was used to measure visuo-proprioceptive alignment precision (σ_MJ_), corresponding to the variability in the localization of the position in space perceived as aligned with the body midline, and visuo-proprioceptive alignment accuracy (MJ absolute error), corresponding to the angular difference between the perceived and the real localization of the body midline along the different trials, in absolute value.

### 2.3 Statistical analysis

Statistical analyses were performed in Rstudio, with R (R Core Team, 2024). Non-parametric statistical tests were applied on not-normally distributed data, as assessed by the Shapiro-Wilk test. Only extreme outliers, defined as values exceeding three-times the interquartile range, were excluded. No other preprocessing step was performed on the data, except for the normalization of reaching errors mentioned above and reported already elsewhere (Bertoni et al., 2023; Risso et al., 2025).

To quantify age-effects on BO, two linear mixed models were fitted (lmm formula: ownership ∼ disparity*age + (1| subject)), one for the explicit condition and one for the implicit condition, with age treated as a continuous variable.

Then, to evaluate whether reaching errors truly reflect explicit ownership as measured by subjective ratings, we computed residual values for both measures. To isolate trial-by-trial variability independent of disparity, residuals were computed by subtracting the mean rating and reaching error at each disparity level. Residual errors were signed according to disparity direction, and their correlation with residual ownership ratings was assessed (excluding 0° trials).

Consistent with previous works (Bertoni et al., 2023; Mastria et al., 2025; Risso et al., 2025), to investigate the bottom-up and top-down contributions underlying BO, we took advantage of the Bayesian CI model. Within this framework, the weight attributed to vision and proprioception (i.e., the bottom-up sensory components) depends on the precision of each sensory modality (σ_P_ and σ_V_), while the top-down contribution on the prior probability of a common cause for the visual and the proprioceptive signals (P_*com prior*_; this includes perceptual priors, such as expectations derived from past sensory experiences, and cognitive priors e.g., more abstract, high-level knowledge).

σ_V_, σ_P_ and P_*com prior*_ were inferred by fitting a Bayesian CI model at the individual level on the VPD multisensory performance using the BADS Matlab optimization tool (Acerbi and Ji, 2017) and simulating 50.000 trials for each spatial disparity.

Since we aimed at investigating also age-related changes in the multisensory processing underlying BO from adolescence to old age, we first run correlation analyses on age and the bottom-up (σ_V_ and σ_P_) and top-down (P_*com prior*_) components extracted from the Bayesian CI model. Then, to confirm the predictions of the model, we correlated participants’ age to their real sensory performance measured the unisensory tasks (σ_PJ_, σ_MJ_, PJ and MJ absolute error). Further analysis supporting the coherence between the extracted and the observed sensory behavior are provided in Supplementary material.

Unfortunately, due to technical issues (i.e., wrong initial configuration of the set-up), data from two young subjects at the PJ and MJ task were not considered for data analysis.

Finally, we assessed whether regardless of age, proprioceptive precision was linked to the subjective experience of ownership toward the virtual hand by correlating the proprioceptive variability at the PJ task (σ_PJ_) with the mean subjective ownership ratings at the explicit VPD.

Mean values and standard deviations for the VPD and the unisensory tasks across age-groups are reported in the Supplementary Tables 1, 2.

## 3 Results

### 3.1 Body ownership across physiological aging: progressive increase in ownership for incongruent virtual hands

Overall, the two lmm, respectively on ownership ratings and reaching errors, showed substantial explanatory value (explicit condition: conditional R2 = 0.69; implicit condition: R2 = 0.42) and the part related to the fixed effects alone was 0.52 and 0.40, respectively. The full models’ results are summarized in Supplementary Table 3.

As expected from previous studies (Bertoni et al., 2023; Risso et al., 2025), in the explicit condition the effect of all non-zero disparities was always significant and negative (β = −7.22 to −4.50; *p* < 0.001), indicating that regardless of their age, subjects reported higher ownership ratings when the virtual hand was congruent to the real hand’s position, and lower ratings when a spatial displacement was applied to the virtual hand. Consistently, the effect of all non-zero disparity levels on implicit ownership was also always significant (negative disparities: β = −3.17 to −1.63; *p* ≤ 0.003; positive disparities: β = 1.16 to 2.17; *p* ≤ 0.039), with larger reaching errors when the virtual hand was spatially incongruent with respect to the real hand’s position.

**FIGURE 2:**
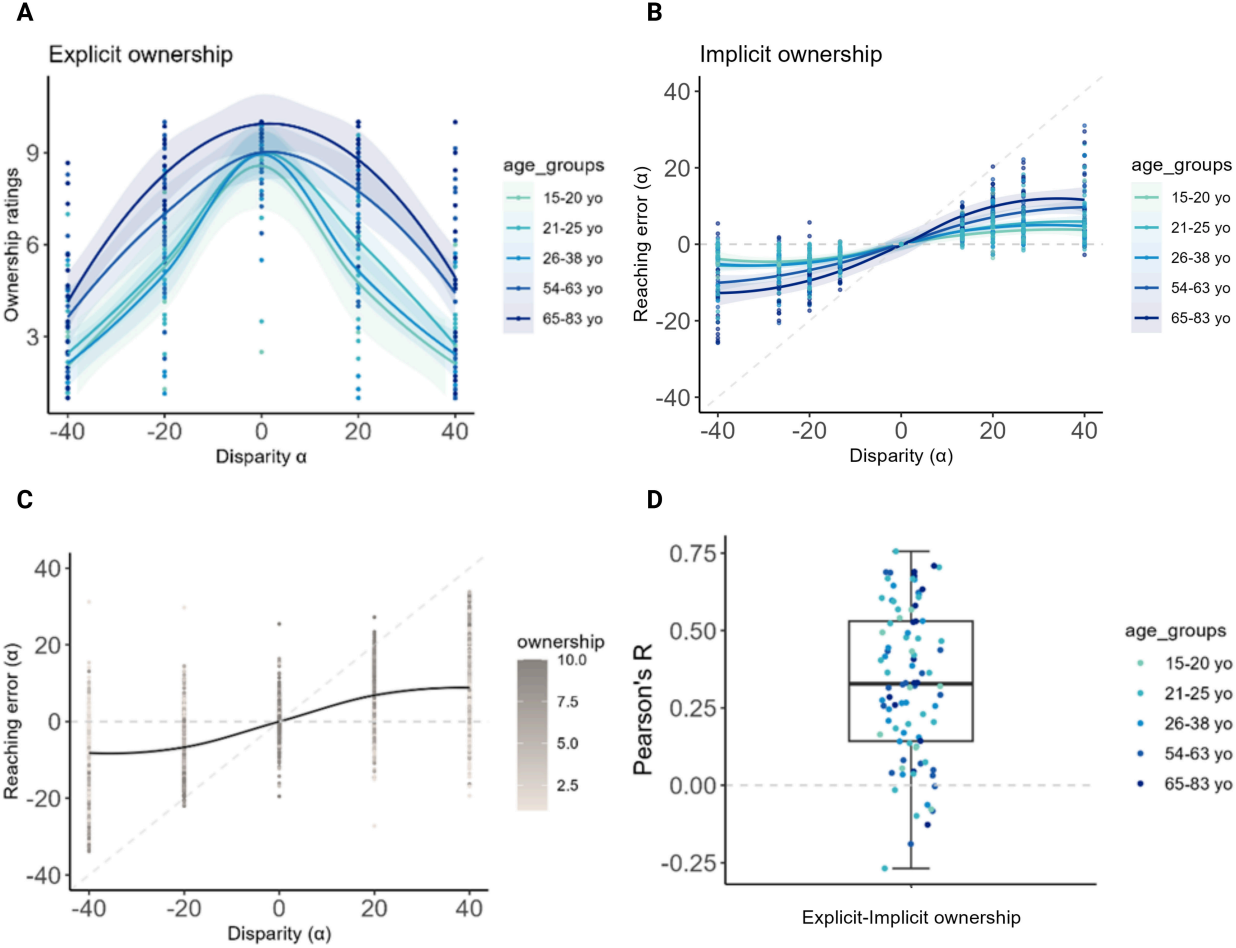
Results at the Visuo-Proprioceptive Disparity task (VPD). **(A,B,D)** A color gradient was used for illustrative purposes to visually represent age along a continuum, with younger participants in light blue and older participants in progressively darker blues (the visual grouping is solely for display, as age was treated as a continuous variable in all the analyses). **(A)** Explicit ownership. The x-axis represents visuo-proprioceptive disparities, and the y-axis shows the average ownership ratings on a scale from 1 (low ownership) to 10 (high ownership). Ownership was highest under the congruent condition (0° disparity); additionally, at increasing age, participants provided significantly increasing ownership ratings to virtual hands displaced with respect to the real hand’s position, at all non-zero disparities. **(B)** Implicit ownership. The x-axis represents visuo-proprioceptive disparities, and the y-axis shows the reaching errors. The horizontal dashed line represents expected errors under pure proprioceptive guidance, while the diagonal dashed line represents expected errors under pure visual guidance. As age increases, reaching errors tend to align to the diagonal line suggesting a progressively higher tendency to follow the visual virtual hand when displaced with respect to the real hand’s position, at all non-zero disparities. **(C)** Relation between explicit and implicit ownership. The plot displays the mean reaching errors at the third block, during which ownership ratings were also collected. Higher ownership ratings, represented by dark gray dots, were reported under the congruent condition and at lower disparities (± 20°) and were accompanied by reduced reaching errors. On the other hand, participants showing higher reaching errors at higher disparities also provided higher ownership ratings. This suggests a relationship between the implicit and the explicit behavior at the VPD task. **(D)** Correlation between explicit and implicit ownership. The plot shows for each participant the Pearson’s correlation coefficients between residual reaching errors and ownership ratings. As visible, the majority of the dots represent a positive correlation. Created in BioRender. Martinelli, I. (2025) https://BioRender.com/l19j3sz.

Crucially, specific effects of age emerged. In the explicit condition, participants showed a tendency to report higher explicit ownership ratings with increasing age (β = 0.02; *p* = 0.055). This was particularly significant when the virtual hand was spatially displaced from the position of the real hand, as indicated by the significant and positive interactions between age and disparities (β = 0.02 to 0.05; *p* < 0.001; Figure 2A).

Significant and positive interactions between age and non-zero disparities were observed also at the implicit condition (negative disparities: β = −0.15 to −0.06; *p* < 0.001; positive disparities: β = 0.07 to 0.14; *p* < 0.001), with reaching movements increasingly shifted towards the virtual hand in older participants (Figure 2B).

Notably, and in line with previous results (Bertoni et al., 2023; Risso et al., 2025), reaching errors showed a trial-level covariance with explicit ownership ratings, suggesting that the reaching errors from the VPD task implicitly reflect the subjective sense of ownership experienced by participants (Figures 2C, D).

### 3.2 From proprioception to vision: reshape of sensory weighting underlying body ownership during physiological aging

**FIGURE 3:**
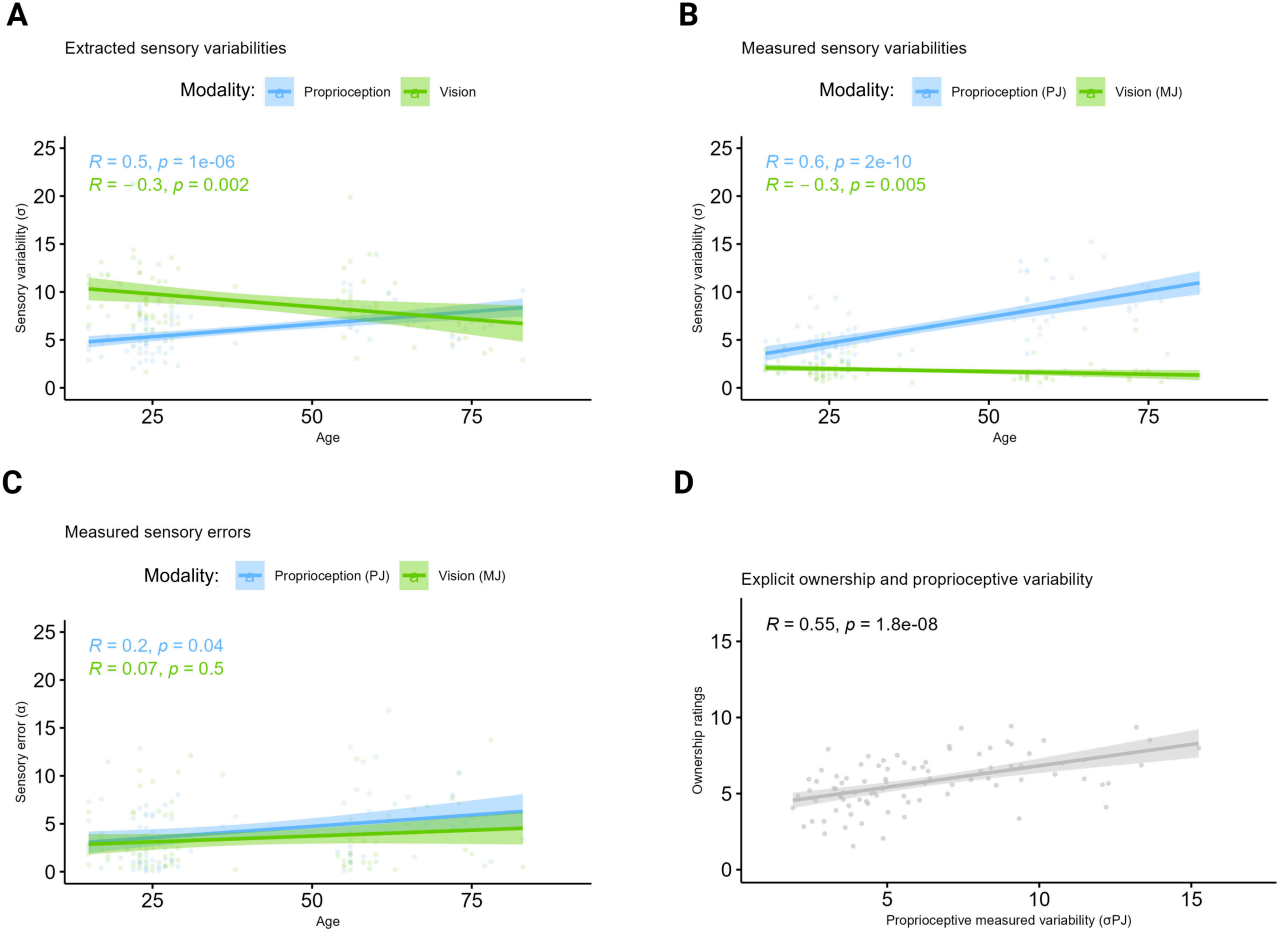
Extracted and measured sensory variabilities underlying ownership. Higher σ values represent higher variabilities and thus lower precision. Higher error values represent lower accuracy. **(A)** Extracted variabilities. Correlation between age, σ_P_ and σ_V_ extracted from the Bayesian CI model applied to the VPD data. The plot shows that age correlated positively with the extracted proprioceptive variability σ_P_ and negatively with the extracted visual variability σ_V_. **(B)** Measured variabilities. Correlation between age, σ_PJ_ and σ_MJ_ measured at the unisensory tasks. The proprioceptive and visual variabilities, respectively measured at the PJ and MJ unisensory tasks showed a similar pattern to that observed from data at the VPD task with a positive correlation between age and the measured proprioceptive variability (PJ), and a negative correlation between age and the measured visual (MJ) variability. **(C)** Measured errors. Correlation between age and errors measured at the PJ and MJ tasks. In terms of unisensory accuracy, the plot shows that with increasing age, only significantly higher proprioceptive errors were observed. **(D)** Correlation between explicit ownership ratings and sigma σ_PJ_. Regardless of their age, participants with higher proprioceptive variability (i.e., lower precision) as measured at the PJ task experienced on average higher ownership for the virtual hand. Created in BioRender. Martinelli, I. (2025) https://BioRender.com/x0lixzb.

Concerning age-related changes in bottom-up and top-down BO components extracted from the VPD task through the Bayesian CI model, results showed that age correlated positively with the extracted proprioceptive variability (σ_P_; rho = 0.50, *p* < 0.001) and negatively with the extracted visual variability (σ_V_; rho = −0.33, *p* = 0.002; Figure 3A). This pattern was consistent with participants’ behavior at the unisensory task PJ, capturing proprioceptive abilities, and MJ, capturing the ability in combining visual information with proprioceptive cues. Specifically, age positively correlated with the measured proprioceptive variability (σ_PJ_; rho = 0.62, *p* < 0.001) and with the absolute proprioceptive error (PJ absolute error; rho = 0.2, *p* = 0.04) at PJ, both indicating a decline in proprioception with a reduction in precision and accuracy occurring with aging (Figures 3B, C). Instead, a negative correlation was found between age and the variability measured at the MJ task (σ_MJ_; rho = −0.30, *p* = 0.005; Figure 3B), further supporting an augmented tendency to rely on vision in older adults, while no significant correlation was found for the absolute error at the same task (*p* = 0.5; Figure 3C). On the other hand, age was not significantly correlated with the top-down component (i.e., prior) extracted from the model (P_com_ _prior_; *p* = 0.1; see Supplementary Figure 1).

To conclude, the averaged subjective ownership ratings and the measured proprioceptive variability at the PJ task positively correlated (rho = 0.55, *p* < 0.001), indicating that regardless of age, the lower was the proprioceptive precision, the higher was the tendency to incorporate the virtual hand as part of one’s own body (Figure 3D).

## 4 Discussion

The feeling that our body belongs to ourselves, commonly referred to as BO, relies on the integration of multisensory signals coming from the world and from the body itself (Bassolino and Serino, 2022; Blanke et al., 2015; Ehrsson, 2020; Tsakiris, 2010). As the human sensory system undergoes significant transformations throughout life, this study aimed at better characterizing age-related changes in hand ownership and the underlying bottom-up and top-down components during the natural aging process from adolescence to old age.

Overall, we found that BO varies from adolescence (age 15) to old age (up to the age of 83), with a higher tendency to incorporate virtual hands spatially incongruent with respect to one’s own hand, as assessed both at explicit (ownership ratings) and at implicit (reaching errors) level. Interestingly, the observed changes in BO appear to be driven by a reshape of the relative weighting of sensory inputs. Specifically, the variabilities extracted by the Bayesian CI model indicate that proprioception serves as the most reliable cue in younger individuals. However, as proprioceptive precision declines with age, visual input increasingly assumes a dominant role specifically in older adults. This pattern was confirmed by participants’ performance at the PJ and MJ unisensory tasks, showing higher proprioceptive precision and accuracy during young age and higher visuo-proprioceptive precision during old age. Hence, in line with previous evidence (Chancel et al., 2018; de Dieuleveult et al., 2017; Kuehn et al., 2018; Risso et al., 2025), the present study supports the view that age-related changes in BO are primarily explained by a decline of unisensory, proprioceptive capacities occurring during physiological aging, rather than by a deterioration of multisensory integration process.

Additionally, we found that individuals with lower unisensory proprioceptive precision, reflected by higher variability in proprioceptive judgments at the PJ task, exhibited a stronger tendency to incorporate the virtual hand as part of their own body. These results further underline that proprioception plays a fundamental role in the subjective experience of BO across different ages. It is important to emphasize that the proprioceptive abilities we refer to have recently been conceptualized under the term of “high-level proprioception” (Héroux et al., 2022, 2024). As in the present protocol, this involves perceptual judgments made across different frames of reference, since participants had to report the location of their hidden limb (i.e., spatial coordinates of where the hand was located relative to the body) into spatial coordinates of where the hand was located in the external visual environment.

Our findings are coherent with those of Weijs et al. (2024) in showing an increase in explicit ownership at increasing multisensory mismatch (i.e., disparities) as age advances, further demonstrating this effect also in the implicit condition that was not assessed in that study. Specifically, with increasing age we observed wider “spatial windows” (de Dieuleveult et al., 2017; Freiherr et al., 2013; Kuehn et al., 2018) for the integration of visual (i.e., the virtual hand) and proprioceptive (i.e., the real hand) information as reflected by higher reaching errors at higher disparities. Moreover, our approach based on the Bayesian CI model allowed us to clarify the mechanisms underlying the observed changes in ownership and thus demonstrate that they are linked to a decrease in proprioception in older subjects. This empirically supports this same hypothesis proposed by Weijs et al. (2024).

We suggest that the reshape of sensory reliance may reflect a potential compensatory mechanism that naturally occurs during healthy aging. Previous studies have described a recalibration of sensory reliabilities in the context of multisensory integration underlying object perception during development (Gori et al., 2008) and along the lifespan (Gori, 2015). Here, we show that the gradual decline in proprioceptive precision occurring with advancing age is accompanied by a recalibration of sensory weighting in favor of vision that may be crucial for maintaining a coherent sense of BO and effective interactions with the environment. This also aligns with converging evidence even beyond the domain of embodiment, indicating that older adults show reduced weighting of motor and bodily signal in favor of a compensatory increase in visual signal processing, as recently emphasized by Costello and Aho (2025), Costello and Bloesch (2017), Kuehn et al. (2018).

Importantly, while this sensory recalibration was observed in the absence of any clinical condition or overt alteration in BO, it is coherent with previous evidence from pathological populations, which suggests that proprioceptive deficits are associated with disorders of BRs (Garbarini et al., 2020; Romano and Maravita, 2019). Consistent with this perspective Mastria et al. (2025) recently applied the same VPD paradigm on stroke patients, demonstrating BO alterations characterized by an increased tendency to incorporate incongruent virtual hands in patients versus age-matched controls, associated with proprioceptive deficits and damage in the frontoparietal network. Crucially, and contrary to healthy older adults, these patients also showed reduced subjective ownership for their own hand (i.e., virtual hands spatially congruent to their real hand’s position).

Finally, the VPD battery implemented in the current study enabled a comprehensive, quantitative evaluation of age-related changes in the implicit and explicit dimensions of ownership while assessing the underlying bottom-up and top-down components and providing a sensitive assessment of proprioception. This allowed to overcome the well-known methodological limitations of previous experimental paradigms for the study of BO i.e., the RHI (Riemer et al., 2019), which yielded heterogeneous results across subjects of varying ages (Cowie et al., 2013; de Klerk et al., 2021; Filippetti and Crucianelli, 2019; Graham et al., 2015; Lee et al., 2021; Marotta et al., 2018; Palomo et al., 2018), and to support the implementation of more ecological and technology-based proprioceptive evaluation (Salerno et al., 2025). This approach may also inspire the development of targeted tools for the rehabilitation of sensorimotor and BRs deficits in healthy and clinical populations (Risso et al., 2022; Risso and Bassolino, 2022; Bassolino et al., 2022; Crema et al., 2022). The mean values and standard deviations for the VPD and the unisensory tasks across different age-groups provided in Supplementary material could serve as initial normative data for future studies using this paradigm.

This study presents some methodological limitations. First, due to practical factors related to participant recruitment only healthy female participants were included, which may limit the generalizability of the findings to male populations although to date no conclusive evidence on gender-related difference in ownership has been reported (Brizzi et al., 2024; Guerra et al., 2019). Second, certain age groups were not well represented (i.e., middle aged from 40 to 50 years). Future research should aim to address these limitations by extending to even broader age groups, while longitudinal study designs should be implemented to further investigate whether changes in BO may predict functional outcomes such as sensorimotor abilities decline in older adults.

## Data Availability

The original contributions presented in this study are included in this article/Supplementary material, further inquiries can be directed to the corresponding author.
