# Supplementary material for: From adolescence to old age: how sensory precision shapes body ownership during physiological aging
Source: Front Hum Neurosci. 2025 Oct 9;19:1663505. doi: 10.3389/fnhum.2025.1663505 (PMC12547696; doi:10.3389/fnhum.2025.1663505)
Supplement: Supplementary file 1 [file Presentation_1.pdf]

Supplementary Material

1 Application of the Bayesian Causal Inference model to the VPD task

Bertoni and colleagues (1–3) recently validated a Bayesian Causal Inference (Bayesian CI) model of body ownership as arising from the optimal integration of multisensory signals applied to the VPD task. Within this framework, multisensory integration, combining visual, proprioceptive, and other inputs, allows the brain to resolve sensory conflicts and generate unified bodily perceptions. This integration is computationally "optimal", meaning that it produces the lowest-variance estimate.

For visuo-proprioceptive integration, Bayesian theory posits that to interpret a stimulus S_{VP}, the brain computes a weighted average of visual (S_V) and proprioceptive (S_P) signals, with higher weights assigned to the most reliable sensory cue:

$$S_{VP} = w_V S_V + w_P S_P$$

In this equation, the visual weight (w_V) and the proprioceptive weight (w_P) add up to unity ($w_V + w_P = 1$) and are proportional to the reliability R of each cue, where reliability is defined as the inverse of the noise of the corresponding cue ($R_i = \frac{1}{\sigma_i^2}$).

$$w_V = \frac{R_V}{R_V + R_P} \; ; \; w_P \frac{R_P}{R_P + R_V}$$

Hence, assuming the unimodal cues are independent, normally distributed, and derive from a single common source (i.e., forced fusion; FF), the optimal estimation of the hand position (X_{FF}) based on visual and proprioceptive signals is:

$$x_{FF} = x_V \frac{R_V}{R_V + R_P} + x_P \frac{R_P}{R_P + R_V}$$

The final hand position estimate (X_{FF}) merges uncertain visual (x_v) and proprioceptive (x_p) signals weighted by their reliability (σ_V, σ_P). This "forced fusion" estimate assumes full certainty that both inputs come from the same hand. Instead, as the brain first infers the probability (P_{com}) that a seen object (i.e., the virtual hand) is one own's hand, the weighting of sensory cues depends not only on their reliability, but also on the probability P_{com} that the final estimate corresponds to one's own hand. Therefore, based on the Bayesian CI (x_{BCI}) the equation that describes the final estimate of hand position is:

$$x_{BCI} = P_{com} x_{FF} + (1 - P_{com}) x_P$$

The probability P_{com} that a given object (such as the virtual hand) is perceived as one's own hand ranges from 1, indicating complete certainty that the virtual hand belongs to oneself, to 0, indicating complete certainty that it does not. In the model, changes in P_{com} primarily depend on the spatial difference between the virtual hand's position and the hand's location as perceived through proprioception.

$$P_{com} = f(|\, x_V \; - \; x_P|)$$

This function clearly shows that P_{com} depends on the visuo-proprioceptive disparity, represented by $|x_V - x_p|$. Therefore, the model predicts that P_{com} diminishes as the mismatch between visual and proprioceptive inputs increases, while it is maximal under zero visuo-proprioceptive disparity. Moreover, when the visual and proprioceptive precision are low, larger discrepancies are required for P_{com} to show a substantial decline. Finally, larger prior common cause is accompanied by larger P_{com} at all disparities, resulting in a higher tendency to follow the virtual hand during reaching. Concerning the sensory visual and proprioceptive contributions, larger reaching errors are expected in the presence of visuo-proprioceptive incongruence and/or higher explicit ownership ratings are expected (higher values of P_{com}) if more weight is attributed to the visual signal (i.e. the virtual hand) than to the proprioceptive one. Conversely, and coherently with what participants are instructed to do, if proprioception is weighted more than vision then reaching errors should be minimal.

2 Absence of significant correlation between age and the extracted prior

As mentioned in main text, we also investigated age-related changes in the top-down components underlying BO extracted from the Bayesian CI model (i.e., the extracted prior, including perceptual priors like expectations derived from past sensory experiences, and cognitive priors e.g., more abstract, high-level knowledge). Results showed no significant correlation between age and $P_{com\ prior}$ ($p = 0.1$; Supplementary Figure 1).

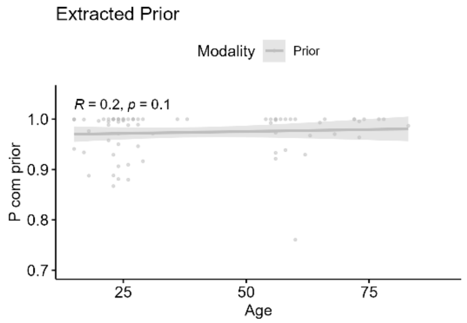

Supplementary Figure 1. Absence of significant correlation between age and the extracted prior.

3 Coherence between sensory precision extracted by the Bayesian CI model and measured at the unisensory tasks

Besides the analyses reported in the main text, to further assess the coherence between the predictions of unisensory variabilities extracted with the Bayesian CI model and the real unisensory proprioceptive and visuo-proprioceptive alignment behaviour of participants, we correlated the extracted sensory precision (σ_P, σ_V) with the proprioceptive and visuo-proprioceptive alignment precision measured with three independent unisensory tasks assessing these abilities (σ_{PJ}, σ_{OLR}, σ_{MJ}).

As represented in Supplementary Figure 2, a significant and positive correlation was found between the extracted proprioceptive variability (σ_P) and the measured proprioceptive variability at the PJ task ($rho = 0.55$; $p < .001$) and at the OLR task ($rho = 0.28$, $p = 0.008$), while no correlation was found between the extracted visual variability (σ_v) and the visuo-proprioceptive alignment variability at the MJ task ($p = 0.546$). These results suggest that the proprioceptive behavior at the multisensory task

truly reflects the sensory variability observed during the two unisensory proprioceptive tasks (PJ and OLR). On the other hand, the absence of correlation between visual behavior at the VPD and the visuo-proprioceptive alignment variability observed during the MJ task may reflect the fact that other different sensory aspects are implicated in the execution of this task.

A B C

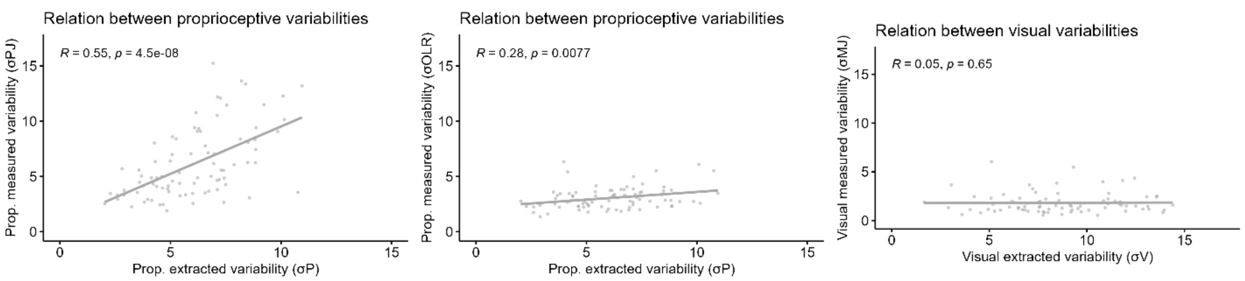

Supplementary Figure 2. (A) Correlation between σ_P and σ_{PJ}. Regardless of age, the proprioceptive variability extracted from the Bayesian CI model fitted on the VPD multisensory data (σ_P) reflected the real proprioceptive behaviour measured at the PJ task (σ_{PJ}). **(B) Correlation between σ_P and σ_{OLR}.** A positive, although weaker, correlation was also found for the real proprioceptive behaviour measured at the OLR task (σ_{OLR}) and the proprioceptive variability extracted from the Bayesian CI model fitted on the VPD multisensory data (σ_P). **(C) Absence of correlation between σ_V and σ_{MJ}.** No relationship was found between the extracted (σ_V) and the measured visuo-proprioceptive alignment precision (σ_{MJ}).

4 Mean values and standard deviations for the VPD and the unisensory tasks across age-groups

In order to provide female initial normative data for the VPD battery, participants were divided into five age groups of balanced size: 15–20 years (N = 10), 21–25 years (N = 27), 26–38 years (N = 20), 54–63 years (N = 20), and 65–83 years (N = 15). Mean values and standard deviations for the VPD task, including reaching errors and explicit ownership ratings, are provided in Supplementary Table 1. See Supplementary Table 2 for proprioceptive and visuo-proprioceptive alignment precision measured at the three unisensory tasks.

**EXPLICIT AND IMPLICIT OWNERSHIP**					
Age groups	*Disparity*	*Ownership ratings*	*Ownership ratings (sd)*	*Reaching errors*	*Reaching errors (sd)*
15-20 yo	-40°	2.05	1.57	-3.81	5.38
	-26.667°	-	-	-4.34	5.20
	-20°	5.29	3.03	-4.81	5.58
	-13.333°	-	-	-3.01	4.51
	0°	8.57	2.25	0.00	3.62
	13.333°	-	-	2.66	4.78
	20°	4.76	3.20	2.76	6.48
	26.667°	-	-	3.95	5.82
	40°	2.11	1.72	3.74	7.09

EXPLICIT AND IMPLICIT OWNERSHIP					
Age groups	*Disparity*	*Ownership ratings*	*Ownership ratings (sd)*	*Reaching errors*	*Reaching errors (sd)*
21-25 yo	-40°	2.46	2.08	-5.55	8.37
	-26.667°	-	-	-5.01	6.66
	-20°	5.46	2.77	-5.25	6.19
	-13.333°	-	-	-3.30	4.76
	0°	9.00	1.82	0.00	3.72
	13.333°	-	-	3.04	4.60
	20°	6.14	2.69	5.70	6.21
	26.667°	-	-	4.95	6.55
	40°	2.73	2.30	5.98	8.74
26-38 yo	-40°	2.12	1.68	-5.12	7.71
	-26.667°	-	-	-5.38	6.43
	-20°	5.04	2.46	-5.41	6.51
	-13.333°	-	-	-3.38	5.13
	0°	8.96	1.40	0.00	4.26
	13.333°	-	-	3.08	5.21
	20°	5.14	2.76	5.62	6.96
	26.667°	-	-	3.89	6.74
	40°	2.43	2.00	4.92	8.41
54-63 yo	-40°	3.55	2.68	-10.10	11.50
	-26.667°	-	-	-7.46	9.33
	-20°	7.01	2.70	-8.27	8.20
	-13.333°	-	-	-4.14	7.27
	0°	9.01	1.37	0.00	5.40
	13.333°	-	-	4.68	6.16
	20°	7.74	2.37	8.18	7.79
	26.667°	-	-	8.04	8.05
	40°	4.39	3.05	9.70	11.47
65-83 yo	-40°	4.10	2.99	-12.76	13.61
	-26.667°	-	-	-11.42	9.36
	-20°	8.27	2.39	-9.66	8.61
	-13.333°	-	-	-7.26	6.27
	0°	9.94	0.27	0.00	5.34
	13.333°	-	-	6.56	6.60
	20°	8.83	2.15	10.83	7.38
	26.667°	-	-	11.18	9.59
	40°	4.82	3.53	11.51	13.84

Supplementary Table 1. Mean values and standard deviation of ownership ratings and reaching errors according to age, at each level of disparity. Ownership ratings were assessed on a scale from 1 to 10; reaching errors were computed as the angular difference between the target position and the real hand's position and were cleaned by subtracting the mean reaching error at 0° disparity from the observed reaching error at the other disparities.

**Unisensory precision extracted and measured**										
Age groups	σ_P	σ_P *(sd)*	σ_V	σ_V *(sd)*	σ_{PJ}	σ_{PJ} *(sd)*	σ_{MJ}	σ_{MJ} *(sd)*	σ_{OLR}	σ_{OLR} *(sd)*
15-20 yo	4.83	1.36	10.53	2.04	3.85	1.29	2.37	0.96	3.10	1.45
21-25 yo	5.02	1.75	9.15	3.76	4.37	1.73	1.87	1.07	3.04	0.73
26-38 yo	5.58	1.98	9.81	4.19	4.73	1.73	2.03	0.91	2.78	0.84
54-63 yo	8.02	1.64	9.93	3.61	8.95	2.89	1.32	0.65	3.45	1.17
65-83 yo	6.66	1.53	5.13	1.30	9.26	3.16	1.82	1.43	2.73	0.58

Supplementary Table 2. Mean values and standard deviations of unisensory precision parameters extracted from the Bayesian CI model (σ_P *and* σ_V) and measured at the unisensory tasks (σ_{PJ}, σ_{MJ}, σ_{OLR}). Higher variability (σ) corresponds to lower precision.

5 Complete results of the linear mixed models for explicit and implicit ownership

The complete output of the two linear mixed model models run for explicit (formula: ownership rating ~ disparity*age + (1| subject)) and implicit (formula: reaching error ~ disparity*age + (1| subject)) are summarized in Supplementary Table 3.

**EXPLICIT OWNERSHIP**							
Fixed effects	*β*	*95% CI*	*df*	*t*	*p*	*Std. β*	*95% CI*
Disparity -40°	-7.06	[-7.52, -6.59]	3228	-29.92	< .001	-1.80	[-1.86, -1.74]
Disparity -20°	-4.53	[-4.97, -4.09]	3228	-20.02	< .001	- 0.85	[-0.91, -0.79]
Disparity 20°	-4.50	[-4.94, -4.05]	3228	-19.87	< .001	-0.74	[-0.80, -0.68]
Disparity 40°	-7.22	[-7.66, -6.78]	3228	-31.91	< .001	-1.67	[-1.73, -1.61]
Age	0.02	[$-3.28e^{-04}$, 0.03]	3228	1.92	0.055	0.09	[$-1.94e^{-03}$, 0.18]
Disparity -40° * Age	0.02	[0.01, 0.03]	3228	4.00	< .001	0.13	[0.06, 0.19]
Disparity -20° * Age	0.04	[0.03, 0.05]	3228	7.88	< .001	0.24	[0.18, 0.30]
Disparity 20° * Age	0.05	[0.04, 0.06]	3228	9.69	< .001	0.29	[0.23, 0.35]
Disparity 40° * Age	0.04	[0.03, 0.05]	3228	7.20	< .001	0.22	[0.16, 0.28]
**IMPLICIT OWNERSHIP**							
Fixed effects	*β*	*95% CI*	*df*	*t*	*p*	*Std. β*	*95% CI*
Disparity -40°	-1.63	[-2.66, -0.61]	12217	-3.14	0.002	-0.77	[-0.81, -0.72]
Disparity -26.667°	-2.32	[-3.42, -1.22]	12217	-4.12	< .001	-0.68	[-0.73, -0.62]
Disparity -20°	-3.17	[-4.58, -1.77]	12217	-4.43	< .001	-0.68	[-0.75, -0.62]
Disparity -13.333°	-1.68	[-2.78, -0.57]	12217	-2.98	0.003	-0.42	[-0.48, -0.37]
Disparity 13.333°	1.35	[0.25, 2.46]	12217	2.41	0.016	0.40	[0.35, 0.46]
Disparity 20°	2.17	[0.77, 3.58]	12217	3.03	0.002	0.70	[0.63, 0.76]
Disparity 26.667°	1.16	[0.06, 2.27]	12217	2.06	0.039	0.65	[0.59, 0.70]
Disparity 40°	1.74	[0.76, 2.72]	12217	3.48	< .001	0.74	[0.70, 0.79]
Age	$1.99e^{-04}$	[-0.02, 0.02]	12217	0.02	0.985	$4.19e^{-04}$	[-0.04, 0.04]
Disparity -40° * Age	-0.15	[-0.17, -0.12]	12217	-12.62	< .001	-0.31	[-0.36, -0.26]
Disparity -26.667° * Age	-0.11	[-0.13, -0.08]	12217	-8.44	< .001	-0.23	[-0.28, -0.17]
Disparity -20° * Age	-0.09	[-0.12, -0.06]	12217	-5.48	< .001	-0.19	[-0.25, -0.12]
Disparity -13.333° * Age	-0.06	[-0.09, -0.04]	12217	-4.82	< .001	-0.13	[-0.18, -0.08]
Disparity 13.333° * Age	0.07	[0.04, 0.09]	12217	5.13	< .001	0.14	[0.09, 0.19]
Disparity 20° * Age	0.12	[0.09, 0.15]	12217	7.26	< .001	0.25	[0.18, 0.31]
Disparity -26.667° * Age	0.13	[0.11, 0.16]	12217	10.18	< .001	0.27	[0.22, 0.33]
Disparity 40° * Age	0.14	[0.12, 0.16]	12217	12.34	< .001	0.29	[0.25, 0.34]

Supplementary Table 3. Summary of the linear mixed models predicting body ownership in the explicit and implicit condition including the predictors disparity and age, and the interaction between these. In both models, disparity was treated as a factor variable and age as a continuous variable.

6 Supplementary references

1. Bertoni T, Mastria G, Akulenko N, Perrin H, Zbinden B, Bassolino M, et al. The self and the Bayesian brain: Testing probabilistic models of body ownership through a self-localization task. Cortex. 2023 Oct;167:247–72.

2. Mastria G, Bertoni T, Perrin H, Akulenko N, Risso G, Akselrod M, et al. Body ownership alterations in stroke emerge from reduced proprioceptive precision and damage to the frontoparietal network. Med. 2025 Apr;6(4):100536.

3. Risso G, Bieri M, Bertoni T, Martinelli I, Mastria G, Catinari L, et al. Proprioception impacts body perception in healthy aging – insights from a Psychophysical and Computational Approach. iScience. 2025;113481.
